# Hépatite auto-immune déclenchée après une hépatite aiguë grave à Epstein Barr virus

**DOI:** 10.11604/pamj.2020.37.77.20817

**Published:** 2020-09-18

**Authors:** Khaoula El Montacer, Wafaa Hliwa, Fz El Rhaoussi, Mohammed Tahiri, Fouad Haddad, Ahmed Bellabah, Wafaa Badre

**Affiliations:** 1 Service d'Hépato-Gastro-Entérologie du Centre Hospitalier Universitaire Ibn Rochd, Casablanca, Maroc

**Keywords:** virus EBV, hépatite auto-immune, thérapie corticostéroïde, Epstein Barr virus, autoimmune hepatitis, corticosteroid therapy

## Abstract

Les hépatites non alphabétiques (Epstein Barr virus -EBV-, Cytomégalovirus -CMV-, Herpes simplex virus -HSV-, Varicelle zona virus -VZV-...) peuvent être un mode de révélation de plusieurs hépatopathies chroniques sous-jacentes dont l'hépatite auto-immune (HAI). Nous rapportons le cas particulier d'une hépatite aiguë à EBV révélant une hépatite auto-immune type I confirmée à la ponction biopsie hépatique, chez une patiente de 29 ans suivie pour une néoplasie mammaire. Patiente âgée de 29 ans, suivie pour un carcinome mammaire prévu pour radio-chimiothérapie, hospitalisée au service pour une hépatite aiguë grave (ictère fébrile, hyper-transaminasémie avec ASAT à 47 la normale et ALAT à 23 la normale et un taux de prothrombine à 25%). Les hépatites virales A, B, C et E étaient négatives et les veines sus-hépatiques étaient libres au Doppler. Une hépatite non alphabétique était suspectée devant l´ictère fébrile et le bilan avait révélé une infection à EBV récente retenue devant des Ac anti-VCA type IgM/G et Ac anti-EBNA type IgG positifs. La patiente était traitée par l´acyclovir pendant 10 jours. L'évolution était marquée par l'apparition d'une ascite et le diagnostic d'une hépatite auto-immune type était retenue consécutivement sur des arguments biologiques (un pic gamma à l´électrophorèse des protéines sériques et des anticorps anti-nucléaires positifs) et histologiques. Une rémission clinico-biologique était obtenue sous corticothérapie. Les infections à EBV doivent être recherchées devant tout tableau d'hépatite aiguë fébrile survenant sur un terrain d´immunodépression. Les praticiens devront également être alarmés à une cytolyse persistante après l'épisode infectieux afin de guetter un déclenchement d'une éventuelle HAI notamment chez le sexe féminin, en cas de contexte d'auto-immunité et devant la négativité des sérologies infectieuses virales alphabétiques.

## Abstract (en)

Non-alphabetical hepatitis (Epstein Barr virus -EBV-, cytomegalovirus -CMV-, Herpes simplex virus -HSV-, varicella zoster virus -VZV-etc.) may be a mode of revelation of several underlying chronic liver diseases including autoimmune hepatitis (HAI). We report a peculiar case of acute EBV hepatitis, revealing type I autoimmune hepatitis confirmed by liver biopsy through puncture in a female patient on breast cancer treatment. The study involved a 29-year-old female patient on breast cancer treatment scheduled to receive radiotherapy and chemotherapy, hospitalized for acute severe hepatitis (fever with jaundice, hypertransaminasemia (normal AST level 47 and normal ALT level 23 and prothrombin activity 25%). The test for viral hepatitis A, B, C, and E was negative and subhepatic veins were free on doppler. Non-alphabetical hepatitis was suspected based on fever with jaundice. Patient’s assessment showed recent EBV infection diagnosed on the basis of the presence of anti-VAC IgM/G and anti-EBNA Ab IgG. The patient received acyclovir for 10 days. Progression was marked by ascites. The diagnosis of autoimmune hepatitis was retained based on laboratory tests (gamma peak on serum protein electrophoresis and positive anti-nuclear antibodies) and histological examination. Clinical-biological remission was obtained with corticosteroid therapy. EBV infections should be investigated in immunocompromised patients with fever in the clinical course of acute hepatitis. Practitioners should also suspect it in patients with persistent cytolysis following an infectious episode in order to prevent the occurrence of autoimmune hepatitis, in particular in female patients, in a context of self-immunity and negative serological tests for alphabetical viral hepatitis.

## Introduction

L´hépatite auto-immune est une maladie rare dont l´étiopathogénie reste mal élucidée faisant intervenir des facteurs génétiques, immunologiques et environnementaux dont essentiellement les agents viraux hépatotropes et non hépatotropes; la famille des Herpes Viridaes est le chef de fil [1, 2]. Nous rapportons l'observation particulière d'une hépatite aiguë sévère à EBV révélant une hépatopathie auto-immune.

## Patient et observation

Il s´agissait d´une patiente âgée de 29 ans, suivie pour un carcinome mammaire diagnostiqué sur une pièce de tumorectomie avec un curage ganglionnaire et prévue pour radio-chimiothérapie. L´anamnèse n´avait pas révélé une notion de prise médicamenteuse hépatotoxique ni de phytothérapie. Aucun contage virale n´était rapporté. La patiente était hospitalisée au service d´hépato-gastro-entérologie du Centre Hospitalier Universitaire du Ibn Rochd de Casablanca pour une hépatite aiguë sévère fébrile, révélée par un ictère cutanéo-muqueux généralisé d´allure cholestatique. L´examen abdominal avait noté une sensibilité épigastrique et de l´hypochondre droit sans signes d´insuffisance hépatocellulaire ni d´hypertension portale ni organomégalie. Avec au bilan biologique, une bilirubine totale élevée à 240mg/l à prédominance conjuguée à 233,9mg/l, une cholestase avec des gamma-GT à 135,9UI/l soit 2,19N, des phosphatases alcalines à 187,9UI/l soit 1,4N associées à une cytolyse hépatique majeure avec des ALAT à 929UI/l soit 18,5N et des ASAT à 1180UI/l soit 26N. Les taux de prothrombine et le facteur V étaient bas à 48%. Le taux d´albumine et la fonction rénale étaient corrects. La numération formule sanguine n´avait pas montré d´hyperleucocytose ni de thrombopénie toutefois une monocytose à 1310e/ul était notée ainsi qu´une légère augmentation de la C-réactive protéine (CRP) à 25,4mg/l.

Une échographie abdominale et une imagerie par résonnance magnétique des voies biliaires réalisées devant une triade de Charcot (ictère fébrile et douloureux) évocatrice d´une angiocholite, avaient éliminé une obstruction des voies biliaires. Les sérologies virales alphabétiques (A, B, C et E) étaient toutes négatives. Devant le terrain de néoplasie pro-thrombogène, l´écho-Doppler des veines sus-hépatiques avait permis d´écarter un éventuel syndrome de Budd-Chiari. Le diagnostic de leptospirose n´était pas soulevé vue l´atteinte cytolytique prédominante, l´absence d´un syndrome hépatorénal, d´une thrombopénie et d'un contexte épidémiologique évocateur. Le tableau clinique survenant à distance de la tumorectomie, l´absence d´un syndrome de Stauffer et la rareté d´une hépatite infiltrante en cas de cancer de sein étaient des arguments rendant le diagnostic d´une hépatite d´origine néoplasique également peu probable.

Les explorations étaient alors complétées, devant le terrain d´immunodépression relative et l´ictère fébrile, par la recherche d´une hépatite non alphabétique. Ainsi le diagnostic d'une hépatite à EBV était retenu sur un profil sérologique d´Ac anti-VCA type IgM/G et Ac anti-EBNA type IgG positifs, respectivement à 24.2, 42 et 197U/ml. L´évolution clinique était marquée par la survenue d´une ascite transudative à 17g/l. Une hépatite auto-immune type I était ensuite retenue sur des arguments biologiques (une hyper-gammaglobulinémie à 24,6g/l et des anticorps antinucléaires (AAN) positifs à 320) et histologiques avec à la ponction biopsie hépatique (PBH), faite après assèchement de l´ascite ramenant une carotte de 8mm renfermant 5 espaces portes, une fibrose septale, une nécrose péri-portale, une empéripolèse, une interface lymphocytaire et une cholestase modérée. Le score de l´IAIHG était à 11.

La patiente était mise sous Acyclovir 10mg/kg/8h pendant 10 jours puis sous corticothérapie à 40mg/j. L´évolution était marquée par une régression progressive de l´ictère et une normalisation des enzymes hépatiques, du taux de la bilirubine et du taux de prothrombine à 2 mois de prednisolone. Vue le contexte néoplasique, l´Azathioprine n´a pu être introduit. La patiente avait complété les séances de chimiothérapie et de radiothérapie sans incidents et est désormais considérée en rémission. Au cours d'une première tentative de dégression rapide des doses de corticothérapie, une ré-ascension des enzymes hépatiques était notée à une dose de 25mg/j de prednisolone. Une réintroduction de la corticothérapie pleine dose avait normalisé le bilan hépatique et aucune rechute n´était survenue au décours d'une deuxième tentative de dégression plus lente après un recul d´un an et demi.

## Discussion

L´Epstein Barr virus est un *gammaherpesvirinae* ubiquitaire infectant 95% de la population mondiale [3]. Quoiqu´il soit un virus peu hépatotrope, une hépatite pourrait survenir de sévérité variable allant de la simple perturbation du bilan hépatique avec une atteinte cytolytique prédominante aux hépatites aiguës graves voire fulminantes tel le cas rapporté par Palanduz *et al* à propos d´une insuffisance hépatocellulaire fulminante associée à une anémie hémolytique sévère liées à une infection à EBV chez une patiente de 7 ans sans antécédents pathologiques notables [4].

Ce virus a été également incriminé dans la survenue de certaines maladies auto-immunes chez des patients génétiquement prédisposés telle l´hépatite auto-immune (HAI), particulièrement chez les malades porteurs de HLA DR3 et DR4. Cette auto-immunité pourrait être provoquée via cinq mécanismes: le mimétisme moléculaire, la stimulation de la lymphoprolifération, le phénomène d'extension des épitopes, la liaison des super-antigènes à des récepteurs des lymphocytes T et CMH2 et l´activation tiers non désirée des cytokines induisant des cellules auto-réactives T [5]. Ainsi une acutisation d'un fond d´hépatopathie chronique par une infection à EBV pourrait être un mode de révélation de la maladie [1] comme notre cas l´illustre. Cependant, compléter les investigations génétiques afin d´identifier le terrain prédisposant à l´auto-immunité, était limité chez notre malade par les moyens financiers. Dans notre observation, l´hépatite auto-immune était retenue sur un score de 11 rassemblé selon les critères de l´IAIHG et l´infection à EBV en était le facteur déclenchant retenue sur un profil sérologique signant une phase transitoire. Elle enrichit une littérature indigente réduite à des rapports de cas (Tableau 1) [6-10] attestant de l´originalité de cette observation.

**Tableau 1:** rapports de cas d’hépatites auto-immunes déclenchées par une infection à EBV

Auteur et bibliographie	Année	Nombre de cas dans l'étude	Cas cliniques
Nobili *et al* [6]	2003	1	Une italienne de 5 ans chez qui une hépatite auto-immune type I à anticorps anti-muscle lisse type actine positifs avait fait suite à une pharyngite à EBV. Son haplotype HLA était A3, A30, B8, B13, Bw4, Bw6, Cw6, DR3, DR13, DRw52, DQ6, DQ2
Saadah *et al* [7]	2013	1	Une patiente saoudienne âgée de 13 ans traitée pour une hépatite auto-immune type I à anticorps anti-mitochondries type II sans lésions des canaux biliaires à la biopsie déclenchée suite à une infection à EBV
Aceti *et al* [8]	1995	1	Une hépatite auto-immune à anticorps anti-muscle lisse type actine et anti-nucléaires positifs, déclenchée trois mois après une mononucléose infectieuse résolue
Vento *et al* [9]	1995	13	Le suivi de 13 patients en bonne santé ayant des membres de famille traités pour une hépatite auto-immune. Sept d'entre eux avaient eu une mononucléose infectieuse à EBV dont deux, avec une prédisposition génétique à l'auto-immunisation, avaient développé une hépatite auto-immune 4 mois après
Wada *et al* [10]	2014	1	Une hépatite auto-immune chez un patient de 61 ans déclenchée par une infection à EBV chronique et active

Ces rapports de cas devraient inciter les praticiens à surveiller un déclenchement d´une pathologie auto-immune et plus précisément une hépatite auto-immune à la suite d'une infection à EBV, notamment chez les sujets prédisposés à savoir le sexe féminin et les patients ayant des antécédents familiaux suivis pour une maladie dys-immunitaire. Le rôle pathogénétique direct du virus devrait être traité avant de démarrer des immunosuppresseurs indiqués devant l´HAI afin d´éviter l´aggravation du processus infectieux [11]. Le pronostic par la suite dépendrait de l´histoire évolutive de l´hépatopathie sous-jacente. La rémission biologique chez notre patiente était obtenue sous corticothérapie après avoir reçu 10 jours d´acyclovir.

## Conclusion

Compte tenu du manque de données scientifiques concrètes corroborant les hypothèses impliquant l´EBV dans le déclenchement d´une hépatite auto-immune et le large écart des connaissances en terme de compréhension du substratum physiopathologique responsable de l´activation du processus auto-immune, le praticien serait sensibiliser à ces diagnostics à travers les expériences publiées de ses collègues afin de mener une prise en charge diagnostique et thérapeutique adéquate et précoce d´une maladie dont le délai de prise en charge est un facteur déterminant d´un pronostic favorable.
